# TFR1 knockdown alleviates iron overload and mitochondrial dysfunction during neural differentiation of Alzheimer’s disease-derived induced pluripotent stem cells by interacting with GSK3B

**DOI:** 10.1186/s40001-024-01677-y

**Published:** 2024-02-06

**Authors:** Tao Kang, Zheng Han, Lijuan Zhu, Bingqing Cao

**Affiliations:** 1 Department of Neurology, Shaanxi Provincial People’s Hospital, Xi’an, 710068 China; 2 Department of Anesthesia, Shaanxi Provincial People’s Hospital, Xi’an, 710068 China

**Keywords:** Alzheimer’s disease, TFR1, GSK3B, Iron overload, Mitochondrial dysfunction

## Abstract

Iron metabolism disorders are implicated in the pathogenesis of Alzheimer’s disease (AD). It was previously reported that transferrin receptor (TFR1) expression was upregulated in AD mouse model. However, the precise biological functions of TFR1 in AD progression remains unclear. Herein, we observed a gradual increase in TFR1 protein expression during the differentiation of AD patient-derived induced pluripotent stem cells (AD-iPS). TFR1 knockdown inhibited the protein expression of ferritin and ferritin heavy chain 1 (FTH1), enhanced the expression of ferroportin 1 (FPN1), and decreased intracellular levels of total iron, labile iron, and reactive oxygen species (ROS). Moreover, TFR1 knockdown improved mitochondrial membrane potential (MMP), increased adenosine triphosphate (ATP) content, downregulated mitochondrial fission proteins, and upregulated mitochondrial fusion proteins. TFR1 knockdown alleviated iron overload and mitochondrial dysfunction in neural cells differentiated from AD-iPS, while TFR1 overexpression showed the opposite results. Additionally, TFR1interacted with glycogen synthase kinase 3 beta (GSK3B) and promoted GSK3B expression. GSK3B overexpression reversed the inhibitory effects of TFR1 knockdown on iron overload and mitochondrial dysfunction in AD-iPS differentiated neural cells. In conclusion, TFR1 knockdown alleviated iron overload and mitochondrial dysfunction in neural cells differentiated from AD-iPS by promoting GSK3B expression. Our findings provide a potential therapeutic target for the treatment of AD.

**Graphical Abstract:**

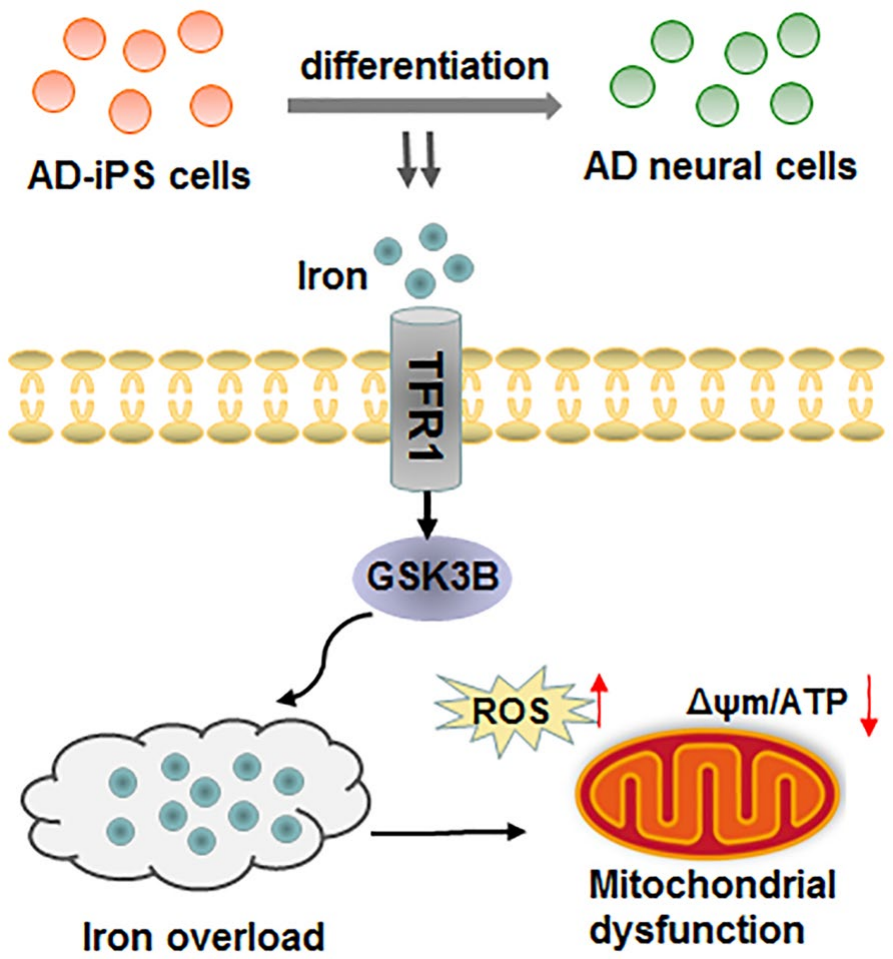

## Introduction

Alzheimer’s disease (AD) is a chronic and progressive neurodegenerative disease and the leading cause of dementia in elderly population [1]. AD patients typically exhibit irreversible cognitive impairments and neuropsychiatric symptoms [2]. The pathology of AD is characterized by the deposition of amyloid plaque, the presence of neurofibrillary tangles (NFTs), and neuronal loss in the brain [3]. Accumulating evidence suggests that extracellular deposition of amyloid β-protein (Aβ) in amyloid plaques and intracellular accumulation of abnormally phosphorylated Tau protein (p-Tau) in NFTs contribute to the development of AD [4, 5]. AD is influenced by various factors, including environment, genetic and epigenetic variations [6]. To date, significant advancement has been made in the diagnosis and treatment of AD. However, current therapies only provide symptomatic relief and do not offer a cure for the disease. Therefore, it is crucial to investigate the molecular mechanisms underlying AD progression.

Iron is an essential element for cellular function and the maintenance of neuronal systems. However, iron metabolism disorders, such as iron overload and iron deficiency, have been observed in various human diseases [7]. In recent years, increasing evidence has indicated that iron metabolism disorder is implicated in the pathogenesis of AD [8, 9]. Disruption of iron homeostasis in the brain can lead to abnormal intracellular iron accumulation, resulting in reactive oxygen species (ROS) production, oxidative stress and inflammatory responses, ultimately leading to cell damage and neurological diseases [10]. Neuronal cells predominantly store iron in the non-toxic form of ferritin, and the storage of iron is tightly regulated by a series of intracellular signaling pathways. Transferrin receptor 1 (TFR1/TFRC) mediates cellular iron uptake, and the number of TFR1 molecules at the cell membrane is associated with iron uptake, thereby contributing to the maintenance of iron homeostasis in neuronal cells and tissues [11]. Upregulated TFR1 leads to excessive iron intake and cellular iron overload [12]. Importantly, previous studies have shown that the mRNA and protein levels of TFR1 are higher in the cerebral cortex of AD mouse model than C57 wild-type mice [13], indicating that the abnormal TFR1 expression may play a role in the development of AD. Moreover, it has been illustrated that apolipoprotein E (ApoE) knock-out induces a progressive iron accumulation with age in the liver and spleen of mice through up-regulating TFR1 and downregulating ferroportin 1 (FPN1), suggesting that ApoE-mediated iron homeostasis is associated with the development of AD [14]. Additionally, iron homeostasis alteration is closely related to mitochondrial dysfunction. Recent studies have demonstrated that impaired mitochondrial function may be associated with cellular iron metabolism disorders and abnormal expression of the membrane protein TFR1 [12, 15].

The discovery and application of human induced pluripotent stem (iPS) cell technology provided a new platform for disease modelling, identification of therapeutic target, and drug discovery. This technology has the potential to explore disease etiology and develop therapeutic strategies [16, 17]. The iPS cells derived from AD patients can be differentiated into disease-relevant cells, such as neurons and glia, which have been used to investigate the underlying pathological mechanisms of AD [18–20].

In this study, we investigated the effects of TFR1 on iron overload and mitochondrial function during iPS cell differentiation, and further explored the regulatory mechanisms involved. We aimed to identify a potential therapeutic target for the treatment of AD.

## Materials and methods

### Brain specimens

Cryopreserved post-mortem brain specimens from AD patients and normal control subjects were obtained from the brain bank at Shaanxi Provincial People's Hospital. The AD group consisted of 14 individuals (10 males and 4 females) with an average age of 80.7 ± 7.3 years, diagnosed based on neuropathology and clinical manifestation. The normal control group included 14 individuals (8 males and 6 females) with non-neurological cognitive impairment or clinical AD presentation. All subjects were enrolled through a prospective donor scheme and provided informed written consent. The study was conducted with the approval of the Ethnic Committee of Shaanxi Provincial People's Hospital.

### Cell lines and culture

The iPS cells derived from peripheral blood monocytes of male AD patients (66540594-1VL), and normal healthy control (66540083-1VL) were obtained from Sigma-Aldrich (St. Louis, MO, USA). Cell culture was performed in reference to a reported method [21]. The iPS cells were cultured in mTesR1 complete medium (STEM CELL Technologies, Vancouver, BC, Canada) diluted with BD Matrigel (BD Biosciences, USA). The culture medium was changed every day. Cells were passaged every 4 days by using 0.5 mM ethylenediaminetetraacetic acid (EDTA) digestion for 3 min. Cells were maintained in a 5% CO_2_ incubator at 37 °C.

### Cell transfection

Overexpression plasmids of TFR1 (pcDNA-TFR1) and glycogen synthase kinase 3 beta (GSK3B) (pcDNA-GSK3B) were custom-synthesized by RiboBio (Guangzhou, China) by subcloning heir full-length cDNAs into pcDNA3.1 vector. Short hairpin RNAs (shRNAs) targeting TFR1 (sh-TFR1 5ʹ-CCA AUA CAG AGC AGA CAU A-3ʹ) and its negative controls (sh-NC; 5ʹ-CAG UCG CGU UUG CGA CUG G-3ʹ) were also synthesized from RiboBio (Guangzhou, China). Lipofectamine 3000 Transfection Reagent (Invitrogen; Thermo Fisher Scientific, USA) was used for cell transfection. In brief, Lipofectamine 3000 reagent (10 μL) was diluted with 250 μL of Opti-MEM solution, and overexpression plasmids/shRNAs were also diluted with 250 μL of Opti-MEM solution. After standing at room temperature for 5 min, these two mixtures were mixed, stood for 20 min, and then added into the cell culture plate when the cell confluence reached 70–80%. The transfection concentration was 2 μg for pcDNA and 1 μg for shRNA. Cells were harvested for further experiments after 48 h of transfection.

### Neural differentiation of iPS cells

Neural differentiation was initiated when iPS cells reached the desired confluence on Matrigel-coated six-well plates by treating them with 1 µM retinoic acid (Sigma-Aldrich, St. Louis, MO, USA) for 4 days based on a reported method [22]. Then, a three-dimensional (3D) iPS cell culture model of AD was established according to a previously reported method [23]. Briefly, RA-induced iPS cells were collected by centrifugation, and suspended in a mixture of BD Matrigel stock solution (BD Biosciences, USA) and neural cell differentiation medium(1:10 volume ratio), and the final cell concentration was approximately 2 × 10^6^ cells/mL. The cell/Matrigel mixture was then transferred into 8-chamber well Lab-Tek II coverglass plates (200 μL/well) and incubated for 1 h at 37 °C to form a thin layer (100–300 nm) 3D gels at the bottom of the plates, and then the media were changed. Subsequently, the medium was changed every 3–4 days. The day of RA induction was designated as day 0 (D0). The differentiation status of iPS cells were evaluated by detecting the expression of neuronal marker protein Nestin at different stages of neural differentiation. The 3D-cultured cells were differentiated for 4–12 weeks depending on the differentiation status.

### Western blot analysis

Cell were incubated with ice-cold RIPA lysis solution (200 µL/well) (Thermo Fisher Scientific, USA) for 20 min, and the cell lysate were centrifuged at 12,000*g* for 5 min to exact total proteins. The protein concentration was quantified using the BCA protein assay kit (Millipore, Billerica, MA, USA). Equal amounts (30 µg) of protein samples were diluted with loading buffer (1:4 volume) and boiled at 95 °C for 5 min. Thereafter, proteins were separated by 10% SDS-PAGE, and then transferred onto polyvinylidene fluoride (PVDF) membranes. After blocking with 5% non-fat milk for 1 h, the PVDF membranes were incubated with primary antibodies overnight at 4 °C, followed by incubation with secondary antibody (1:2000, Abcam, ab6721) for 2 h. Protein signals were detected with an enhanced chemiluminescence detection kit (Pierce Biotechnology, IL, USA) and analyzed with ImageJ software (National Institutes of Health, Bethesda, MD, USA). GAPDH was used as the internal reference. The primary antibodies were obtained from Abcam: anti-TFR1 antibody (1:1000, ab214039), anti-GSK3B antibody (1:5000, ab32391), anti-Aβ antibody (1:1000, ab180956), anti-p-Tau antibody (1:1000, ab92676), anti-Tau antibody (1:1000, ab254256), anti-APP antibody (1:20,000, ab32136), anti-ferritin antibody (1:1000, ab75973), anti-FTH1 antibody (1:1000, ab75972), anti-FPN1 antibody (1:1000, ab58695), anti-DRP1 antibody (1:1000, ab184247), anti-FIS1 antibody (1:10,000, ab156865), anti-MFN2 antibody (1:1000, ab124773), anti-OPA1 antibody (1:1000, ab157457), and anti-GAPDH antibody (1:2500, ab9485).

### Immunofluorescence staining

Cell slides were prepared in culture plates, and washed with PBS buffer for three times. Then, cells were fixed in 4% (w/v) paraformaldehyde for 20 min, permeabilized with 0.1% Triton X-100 in PBS for 20 min, and blocked with 2% bovine serum albumin for 60 min. Cell slides were washed three times with PBS during each operation interval. Then, cells were incubated overnight at 4 °C with primary antibodies: anti-TFR1 antibody (1:100, ab214039), anti-GSK3B antibody (1:100, ab32391), anti-Nestin antibody (1:100, ab105389). The next day, cells were incubated with a secondary antibody conjugated to Alexa Fluor 488 (1:200, ab150157) at room temperature for 2 h. Cell nuclei was counterstained with 1 µg/mL DAPI (D9542, Sigma) for 5 min in the dark at room temperature. Cell images were captured using a fluorescence confocal microscope (Olympus FV3000, Japan) and analyzed ImageJ software (National Institutes of Health, Bethesda, MD, USA).

### Measurement of ROS level

Intracellular ROS level was measured using the ROS detection kit (Beyotime Biotechnology, Shanghai, China) according to the manufactures’ instructions. Briefly, after the indicated transfection, cells cultured in the 6-well plate (1 × 10 ^4^cells/well) were treated with 2ʹ,7ʹ-Dichlorodihydrofluorescein diacetate (DCFH-DA; 10 μM) for 30 min at 37 °C. Next, the fluorescence intensity was measured by using a fluorescence microplate reader (excitation/emission at 495/529 nm; FLx800 Biotek, USA).

### Total iron quantification

Intracellular total iron content was detected using the atomic absorption spectrometer (AAS) (Analytik, Jena, Germany). After the indicated treatment, cells (2 × 10^4^ cells) were harvested by centrifugation and divided into two equal parts. One part was lysed with RIPA lysis buffer to quantify the total protein concentration using a BCA protein assay kit. Another part of cell sample was lysed with 65% HNO_3_ at 70 °C for 2 h, and the cell lysate were collected through centrifugation at 12,000*g* for 5 min at 4 °C for iron quantification. Subsequently, total iron content was measured by AAS and normalized to total protein concentration.

### Labile iron staining

The calcein-acetoxymethyl ester (calcein-AM) method was used to analyze labile iron content as previously described [24]. In brief, cells were collected by centrifugation at 3000*g* for 5 min at 4 °C after the indicated transfection. Then, cells were seeded on a 6-well plate (1 × 10^4 ^cells/well) and then stained with 1 μM calcein-AM and Hoechst 33342 for 30 min at 37 °C in a dark environment. Cell images were captured using a fluorescence confocal microscope under five random fields of view and analyzed ImageJ software (National Institutes of Health, Bethesda, MD, USA). Calcein-AM is a fluorescent probe chelated with ferrous iron. The quenching of calcein-AM fluorescence intensity indicates an increase of chelatable ferrous iron in cells.

### Mitochondrial membrane potential (MMP, Δψm) measurement

MMP alteration was detected by JC-1 staining. After the indicated treatment, cells were seeded on a 6-well plate at a density of 1 × 10^4^cells per well and treated with 5 µM JC-1 at 37 °C for 30 min in the dark. Subsequently, cells were subjected to analysis using a FACS flow cytometer, and the MMP was analyzed by using the FlowJo software.

### Determination of adenosine triphosphate (ATP) content

After indicated treatment, cells were lysed with RIPA lysis solution, and cell lysate were harvested via centrifugation at 12,000*g* for 5 min at 4 °C. Intracellular ATP content was tested with an ATP Determination Kit (Beyotime Biotechnology, Shanghai, China) using a luciferase-based assay according to the manufacturer’s instructions. Briefly, cell lysate and ATP standard solutions were diluted with luciferase buffer, respectively. The luminescence was measured using a Microplate Luminometer (GloMax 96; Promega, USA). Finally, ATP level was calculated based on the standard curve.

### Bioinformatics analysis for protein interaction

Bioinformatics analysis was performed to investigate the potential interaction proteins of TFR1. We searched the potential interaction proteins of TFR1 through three online databases, including GeneMANIA (https://genemania.org/), Biogrid (https://thebiogrid.org/), and Hitpredict (http://www.hitpredict.org/). On the homepage of these websites, we selected homo sapiens, filled in the protein name TFR1, and then clicked “OK” to obtain the potential interaction proteins in the database.

### Co-immunoprecipitation (Co-IP) assay

Cell lysates were harvested by centrifugation and incubated with anti-TFR1 antibody (ab214039, Abcam) or anti-GSK3B antibody (ab32391, Abcam) at 4 °C overnight, followed by incubation with 100 μL of protein A/G agarose beads (Takara Biotechnology, Dalian, China) at 4 °C overnight. IgG (ab172730, Abcam) was used as control. Next, the beads were collected from the mixture through centrifugation at 3000 rpm for 5 min. Following washing with PBS for three times, the beads were boiled with loading buffer for 5 min and then centrifuged at 3000 rpm for 5 min to separate elute the immunoprecipitated proteins. The protein supernatants were harvested by centrifugation and then analyzed with Western blot assay.

### Statistical analysis

The sample size in all experiments was “N = 6”, and each experiment was repeated at least three times. Data are presented as the mean ± the standard error of the mean (SEM) and analyzed using SPSS 22.0 software. The normal distribution of data was accessed using the Shapiro–Wilk test, and the homogeneity of variances was accessed using the Levene’s test. Student’s t-test were used for comparisons between two groups, and one-way analysis of variance (ANOVA) followed by Tukey–Kramer correction was used for the comparison among groups. *P* < 0.05 was considered as statistically significant.

## Results

### Identification of neural cells differentiated from AD-iPS Cells

**Fig. 1:**
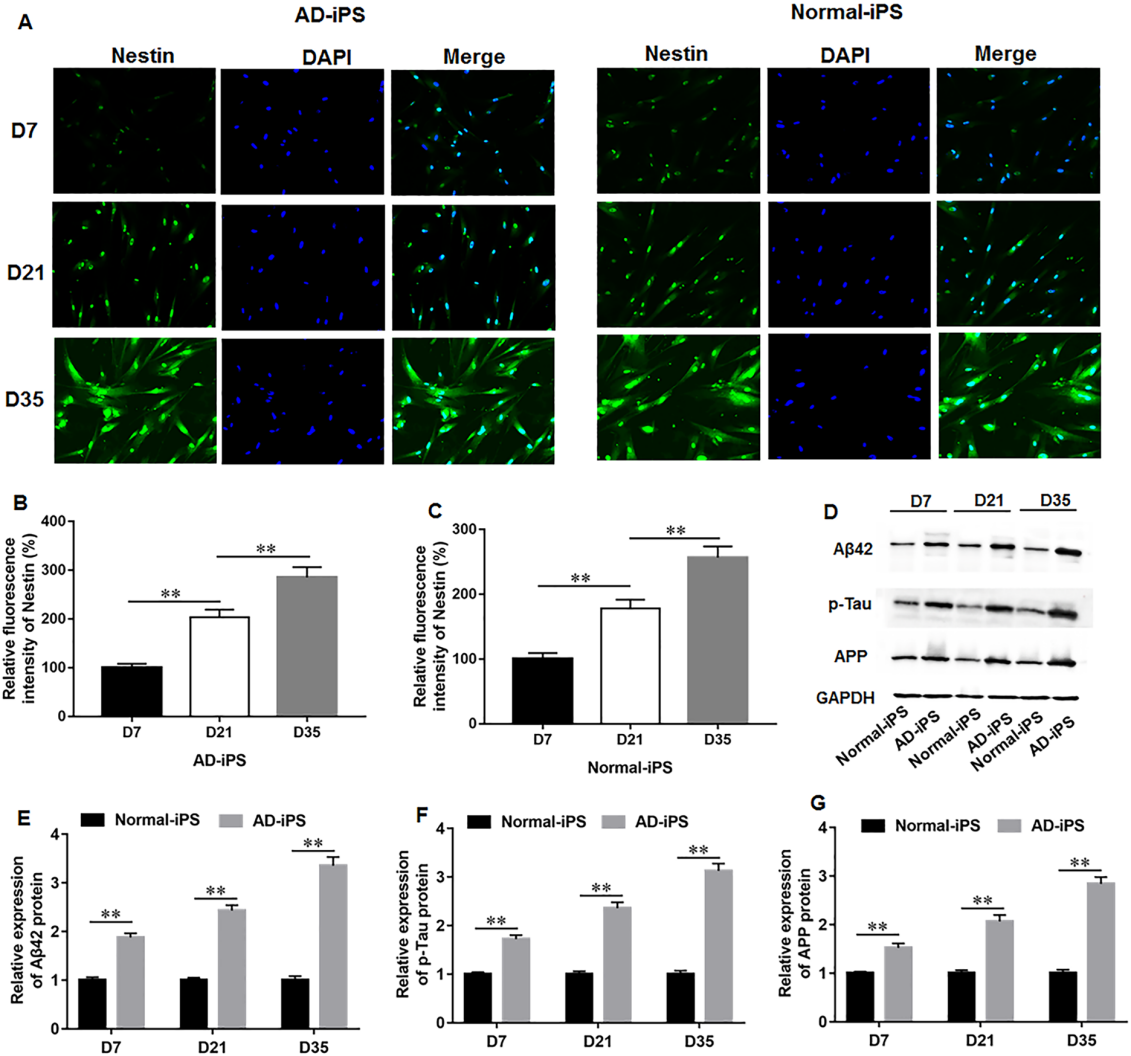
Identification of neural cells differentiated from AD-iPS cells. The iPS cells derived from peripheral blood monocytes of AD patients (AD-iPS) or normal healthy control (Normal-iPS) were treated with retinoic acid and cultured in a 3D human neural cell culture system to induce neural differentiation. **A**–**C** Immunofluorescence staining was performed to access the expression of neural marker protein Nestin on days 7, 21 and 35 of differentiation, respectively. **C**–**F** Western blot assay was used to detect the protein expression of AD pathological marker proteins including Aβ42, p-Tau and APP on days 7, 21 and 35 of differentiation. N = 6. Data from at least three independent experiments were presented as mean ± SEM. **P < 0.01

To establish an accurate human neural cell model of AD, iPS cells derived from peripheral blood monocytes of AD patients (AD-iPS) were treated with retinoic acid and cultured in a 3D human neural cell culture system. The iPS cells derived from normal healthy control (Normal-iPS) were used as control. To identify AD-iPS cell-differentiated neural cells, the expression of neural marker protein Nestin on days 7, 21 and 35 of differentiation were evaluated. The results showed a gradual increase in Nestin expression over time in AD-iPS and Normal-iPS cells, with particularly abundant on day 35, indicating successful differentiation of iPS cells into neural cells (Fig. 1A–C). Moreover, the protein expression of AD pathological marker proteins on days 7, 21 and 35 of differentiation were detected by using Western blot assay. AD-iPS cells exhibited a significant increase in Aβ42, p-Tau, amyloid precursor protein (APP) expression compared to Normal-iPS cells during cell differentiation (Fig. 1D–G), and their levels gradually increased over time. These results confirm the successful establishment of AD neural cells from AD-iPS cells.

### TFR1 was upregulated during AD-iPS cell differentiation

**Fig. 2:**
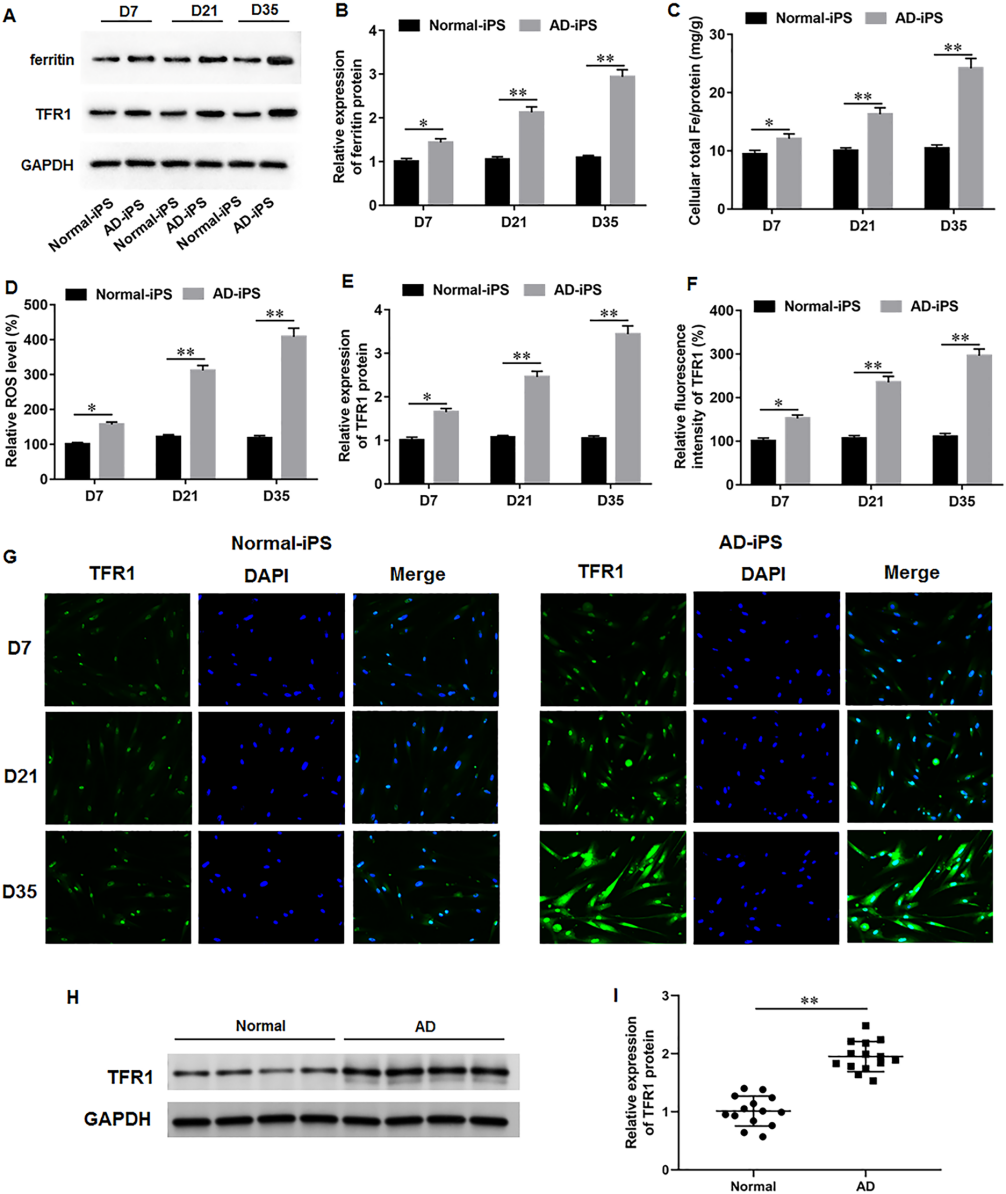
TFR1 was upregulated during AD-iPS cell differentiation. **A**, **B** Western blot assay was performed to measure on days 7, 21 and 35 of differentiation, respectively. **C** Intracellular total iron was evaluated using atomic absorption spectrometer. **D** ROS level was detected using commercial ROS detection kits on days 7, 21 and 35 of differentiation, respectively. **A**, **E** Western blot assay and **F**, **G** immunofluorescence staining were utilized to access TFR1 protein level on days 7, 21 and 35 of differentiation, respectively. **H**, **I** The levels of TFR1 in frontal cortex tissues of AD patients and normal controls were detected using Western blot analysis. N = 6. Data from at least three independent experiments were presented as mean ± SEM. *P < 0.05, **P < 0.01

It was found that iron metabolism dysregulation is associated with the pathogenesis of AD [8]. Subsequently, we investigated the alterations of iron metabolism during the differentiation of AD-iPS cells. Our results illustrated that, compared to Normal-iPS cells, the levels of ferritin (Fig. 2A, B), intracellular total iron (Fig. 2C) and ROS (Fig. 2D) were prominently upregulated during AD-iPS cell differentiation, indicating the occurrence of iron overload. Moreover, Western blot results demonstrated a gradual increase in TFR1 protein expression during AD-iPS cell differentiation compare to Norml-iPS (Fig. 2A, E), which was consistent with the immunofluorescence results (Fig. 2F, G). Additionally, we detected the levels of TFR1 in frontal cortex tissues of AD patients and normal controls using Western blot analysis. The results indicated that TFR1 was upregulated in brain tissues of AD patients compared with normal controls (Fig. 2H, I). Thus, our results demonstrated that TFR1-mediated iron overload might participate in the regulation of AD progression.

### TFR1 knockdown attenuated iron overload and mitochondrial dysfunction during AD-iPS cell differentiation

**Fig. 3:**
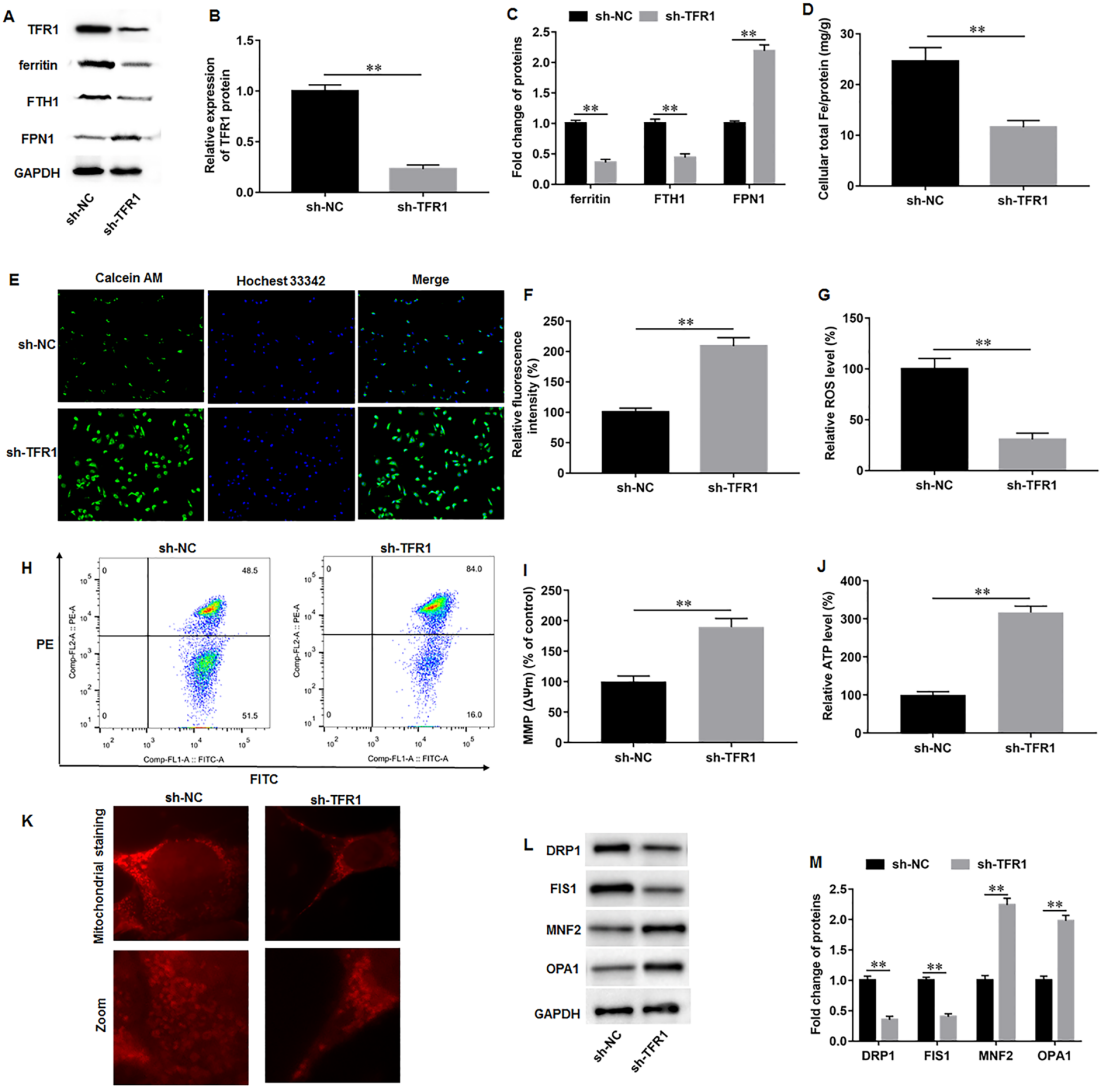
TFR1 knockdown attenuated iron overload and mitochondrial dysfunction during AD-iPS cell differentiation. AD-iPS cells were transfected with sh-TFR1 or sh-NC, and then were induced to differentiate into neural cells. **A**, **B** Western blot assay was performed to access TFR1 level on day 35 of differentiation. **A**, **C** Western blot assay was performed to access ferritin, FTH1, and FPN levels on day 35 of differentiation. **D** Intracellular total iron was evaluated using atomic absorption spectrometer. **E**, **F** Calcein-AM staining was conducted to detect the labile iron pool, and quenching of calcein-AM fluorescence signifies an increase in intracellular labile iron. **G** ROS level was detected using commercial ROS detection kits. **H**, **I** MMP (Δψm) was evaluated by JC-1 staining. **J** ATP content was measured using an ATP determination kit. **K** Mitochondrial staining was used to access mitochondrial fusion and fission. **L**, **M** Western blot assay was used to measure the protein levels of DRP1, FIS1, TFR1, MFN2 and OPA1 were accessed with Western blot assay. N = 6. Data from at least three independent experiments were presented as mean ± SEM. **P < 0.01

We investigated the biological effects of TFR1 knockdown on iron metabolism and mitochondrial function in AD-iPS cell-differentiated neural cells on day 35. Western blot results showed that transfection of sh-TFR1 in AD-iPS cells significantly downregulated TFR1 protein expression compared to transfection of sh-NC (Fig. 3A, B). Ferritin, ferritin heavy chain 1 (FTH1), and FPN1 are crucial proteins associated with iron overload. We found that TFR1 knockdown reduced ferritin and FTH1 levels, and augmented FPN protein expression (Fig. 3A, C). Moreover, TFR1 knockdown decreased intracellular total iron in AD-iPS cell-differentiated neural cells (Fig. 3D). The ferrous iron mainly storages in the labile iron pool, which was determined with Calcein-AM staining. The quenching of calcein-AM fluorescence intensity indicates an increase of chelatable ferrous iron in cells. The results showed that the fluorescence intensity was increased in sh-TFR1 group, suggesting that TFR1 knockdown suppressed the ferrous ion content (Fig. 3E, F). Additionally, TFR1 knockdown decreased ROS generation in AD-iPS cell-differentiated neural cells (Fig. 3G). Besides, we detected the effects of TFR1 knockdown on mitochondrial metabolism. The MMP (Fig. 3H, I) and intracellular ATP content (Fig. 3J) were both increased after TFR1 knockdown. Mitochondrial staining showed that sh-NC group exhibited mitochondrial fission and fragmentation, while sh-TFR1 transfection improved mitochondrial morphology, characterized by typical length and tubular shape (Fig. 3K). Moreover, TFR1 knockdown decreased the levels of mitochondrial fission proteins, dynamic related protein 1 (DRP1) and fission 1 (FIS1), and increased the levels of mitochondrial fusion proteins, mitochondrial fusion proteins mitofusin 2 (MFN2) and optic atrophy 1 (OPA1) (Fig. 3L, M). Collectively, our results revealed that TFR1 knockdown attenuated iron overload and mitochondrial dysfunction during AD-iPS cell differentiation.

### TFR1 overexpression aggravated iron overload and mitochondrial dysfunction during AD-iPS cell differentiation

**Fig. 4:**
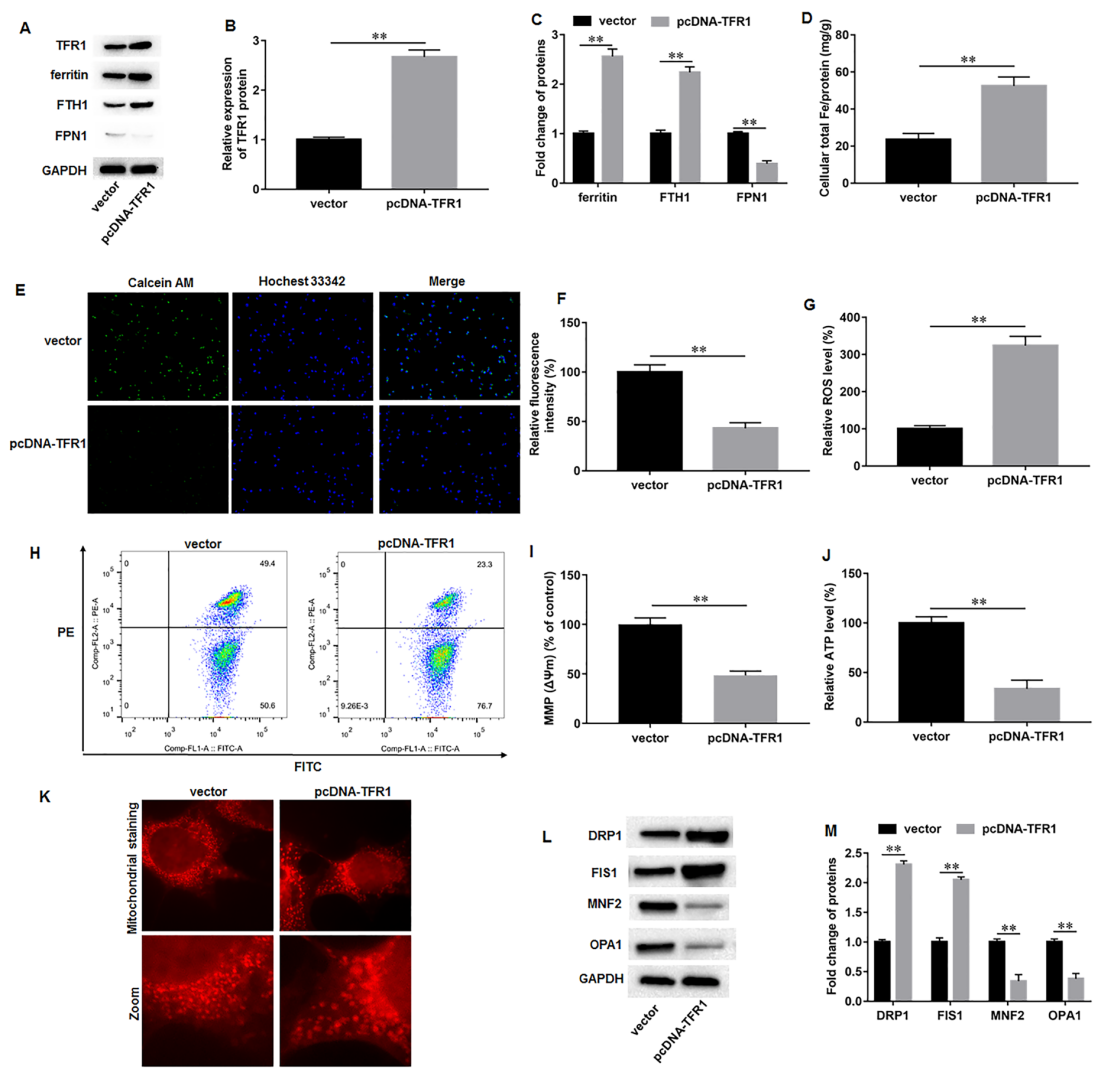
TFR1 overexpression aggravated iron overload and mitochondrial dysfunction during AD-iPS cell differentiation. AD-iPS cells were transfected with pcDNA-TFR1 or empty vector, and then were induced to differentiate into neural cells. **A**, **B** Western blot assay was performed to measure the expression of TFR1 on day 35 of differentiation. **A**, **C** Western blot assay was used to measure the levels of ferritin, FTH1, and FPN on day 35 of differentiation. **D** Atomic absorption spectrometer was performed to detect intracellular total iron content. **E**, **F** Calcein-AM staining was used to detect labile iron pool, and quenching of calcein-AM fluorescence signifies an increase in intracellular labile iron. **G** ROS level was measured by commercial ROS detection kits. **H**, **I** MMP (Δψm) was evaluated by JC-1 staining. **J** ATP content was accessed using an ATP determination kit. **K** Mitochondrial staining was used to access mitochondrial fusion and fission. **L**, **M** Western blot assay was used to measure the protein levels of DRP1, FIS1, TFR1, MFN2 and OPA1. N = 6. Data from at least three independent experiments were presented as mean ± SEM. **P < 0.01

Next, we investigated the effects of TFR1 knockdown on iron metabolism and mitochondrial function in AD-iPS cell-differentiated neural cells. AD-iPS cells were transfected with pcDNA-TFR1 or vector, and then were induced to differentiate into neural cells. Western blot results showed that transfection of pcDNA-TFR1 significantly increased TFR1 protein expression compared to transfection of empty vector (Fig. 4A, B). TFR1 overexpression promoted the protein levels of ferritin and FTH1, and inhibited FPN protein level (Fig. 4A, C). Moreover, TFR1 overexpression augmented the contents of intracellular total iron (Fig. 4D) and labile iron (Fig. 4E, F) in AD-iPS cell-differentiated neural cells. Furthermore, it was observed that TFR1 overexpression enhanced ROS level in AD-iPS cell-differentiated neural cells (Fig. 4G). Additionally, TFR1 overexpression significantly induced MMP/Δψm loss (Fig. 4H, I) and reduced intracellular ATP content (Fig. 4J). Mitochondrial staining showed that the mitochondria in vector group exhibited mitochondrial fission and fragmentation, while pcDNA-TFR1 transfection exacerbated these changes in mitochondrial morphology in AD neural cells (Fig. 4K). Moreover, TFR1 overexpression upregulated DRP1 and FIS1, and downregulated MFN2 and OPA1 (Fig. 4L, M). These results revealed that TFR1 overexpression aggravated iron overload and mitochondrial dysfunction during AD-iPS cell differentiation.

### TFR1 interacted with GSK3B and promoted GSK3B expression

**Fig. 5:**
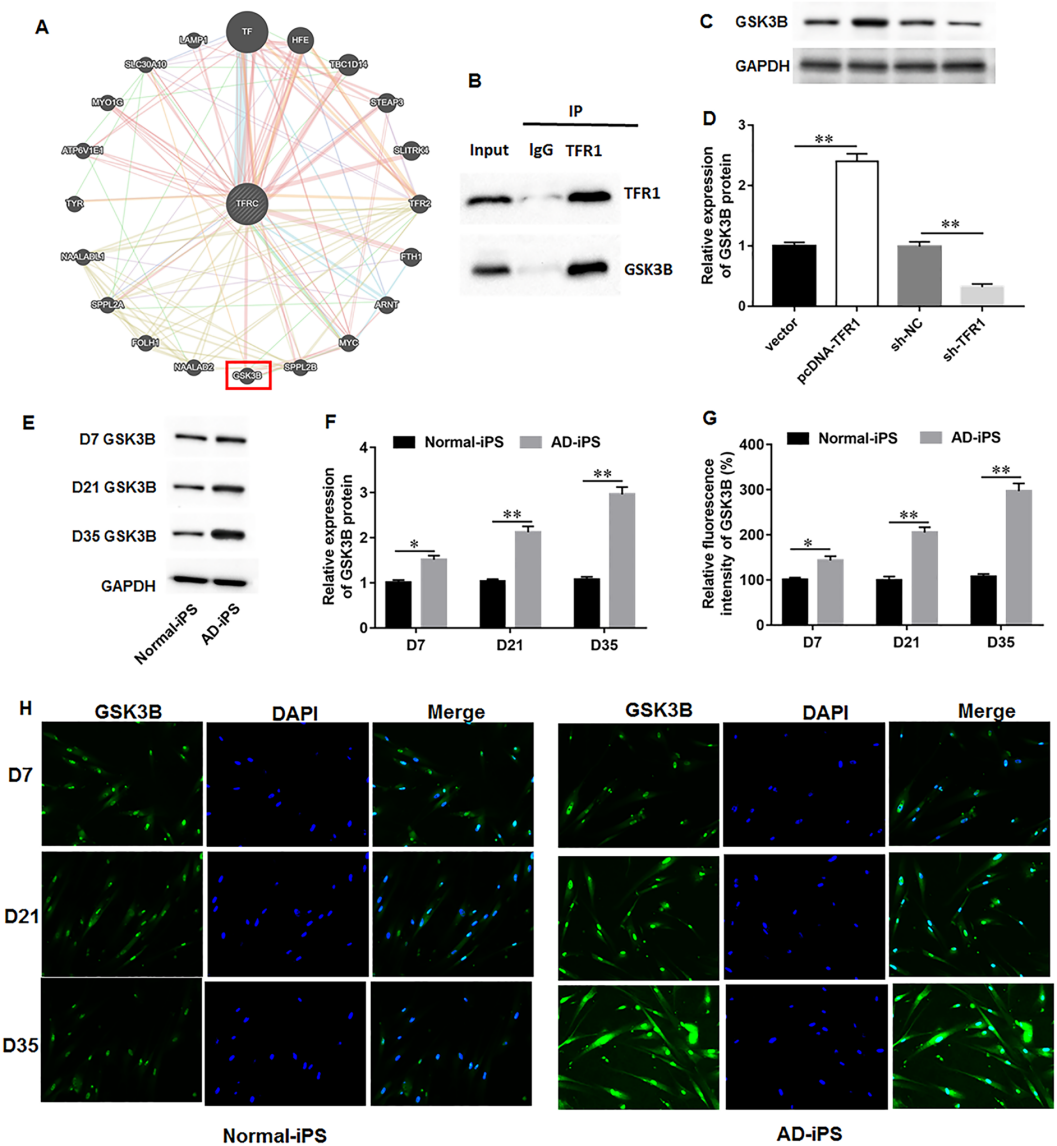
TFR1 interacted with GSK3B and promoted GSK3B expression. **A** The potential interacting proteins of TFR1 were predicted by using the GeneMANIA website (https://genemania.org/). **B** The interaction between TFR1 and GSK3B in AD-iPS cell-differentiated neural cells was verified by using Co-IP assay. **C**, **D** Cells were transfected with pcDNA-TFR1, sh-TFR1, and their negative controls, respectively. GSK3B protein level was accessed with Western blot assay. **E**, **F** Western blot assay and **G**, **H** immunofluorescence staining was employed to access GSK3B protein level on days 7, 21 and 35 of differentiation, respectively. N = 6. Data from at least three independent experiments were presented as mean ± SEM. **P < 0.01

We further investigate the regulatory mechanism of TFR1 in iron metabolism and mitochondrial function during AD progression. It was observed from three online databases including the GeneMANIA, Biogrid, and Hitpredict that there was a potential interaction between TFR1 and GSK3B. The protein interaction image from the GeneMANIA was shown in Fig. 5A. Co-IP assay validated that TFR1 protein could be immunoprecipitated by GSK3B antibody (Fig. 5B), indicating that TFR1 could interact with GSK3B in AD-iPS cell-differentiated neural cells. Next, TFR1 overexpression dramatically upregulated GSK3B protein expression, while TFR1 knockdown restrained GSK3B protein expression (Fig. 5C, D), indicating that TFR1 interacts with GSK3B and positively regulates GSK3B expression in AD-iPS cells. Furthermore, Western blot assay (Fig. 5E, F) and immunofluorescence staining (Fig. 5G, H) confirmed that GSK3B protein expression was gradually elevated during AD-iPS cell differentiation compare to Norml-iPS.

### GSK3B reversed the protective effects of TFR1 knockdown on iron overload and mitochondrial dysfunction during AD-iPS cell differentiation

**Fig. 6:**
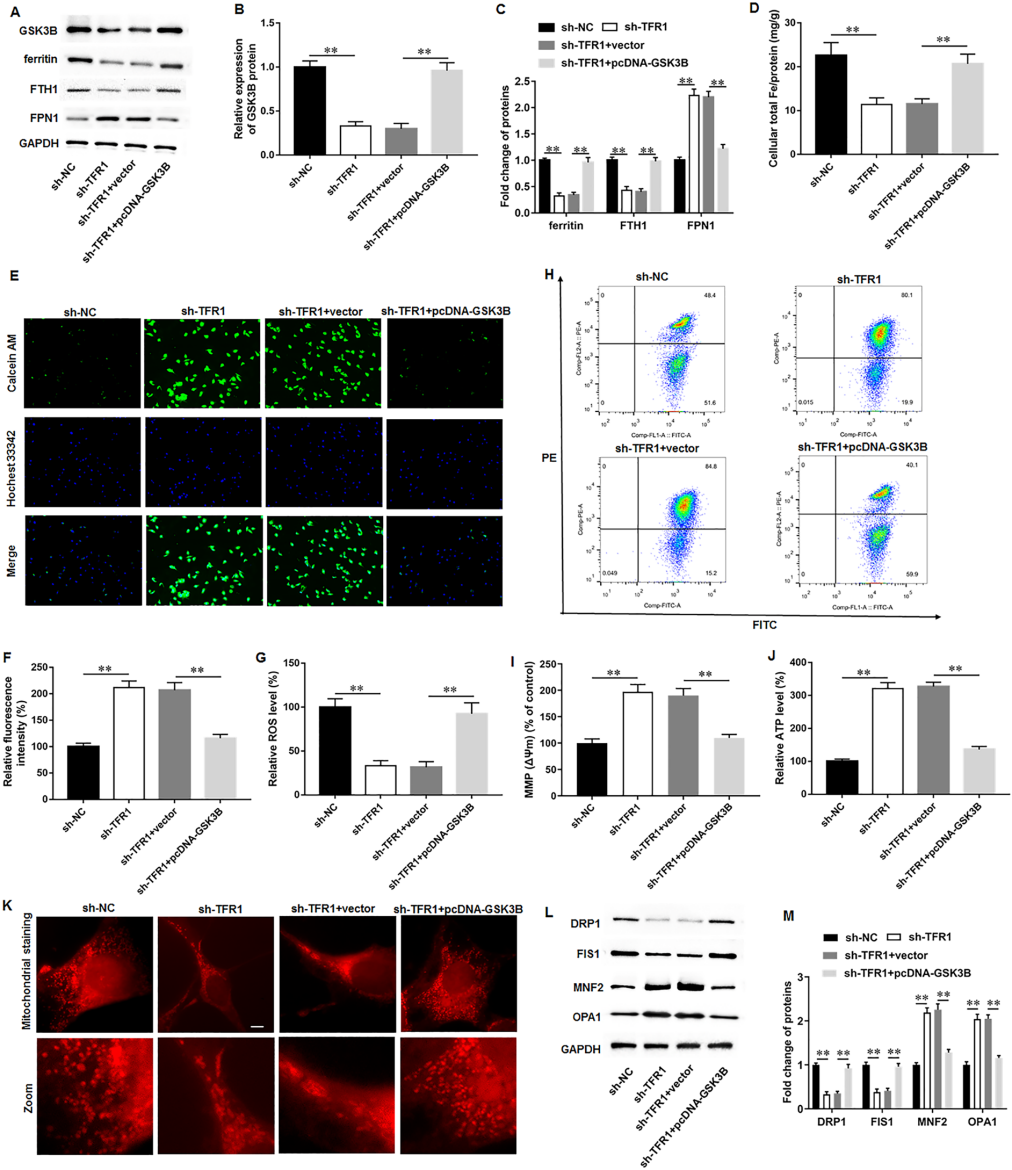
GSK3B reversed the protective effects of TFR1 knockdown on iron overload and mitochondrial dysfunction during AD-iPS cell differentiation. AD-iPS cells were transfected with sh-TFR1 alone or together with pcDNA-GSK3B, and then were induced to differentiate into neural cells. expression, **A**, **B** Western blot assay was performed to examine the protein level of GSK3B on day 35 of differentiation. **A**, **C** Western blot assay was used to evaluate the levels of ferritin, FTH1, and FPN. **D** Atomic absorption spectrometer was used to evaluate intracellular total iron content. **E**, **F** Calcein-AM staining was performed to measure labile iron pool, and quenching of calcein-AM fluorescence signifies an increase in intracellular labile iron. **G** ROS level was examined with commercial ROS detection kits. **H**, **I** MMP (Δψm) was evaluated by JC-1 staining. **J** ATP content was determined using an ATP determination kit. **K** Mitochondrial staining was used to access mitochondrial fusion and fission. **L**, **M** Western blot assay was used to measure the protein levels of DRP1, FIS1, TFR1, MFN2 and OPA1. N = 6. Data from at least three independent experiments were presented as mean ± SEM. **P < 0.01

To further investigate whether TFR1 modulates iron overload and mitochondrial dysfunction in AD progression by regulating GSK3B, AD-iPS cells were transfected with sh-TFR1 alone or together with pcDNA-GSK3B, and then were induced to differentiate into neural cells. We found that the TFR1 knockdown obviously decreased GSK3B expression, while transfection of pcDNA-GSK3B facilitated GSK3B expression in AD-iPS cell-differentiated neural cells (Fig. 6A, B). Then, TFR1 knockdown inhibited ferritin and FTH1 expression, and promoted FPN protein expression, while GSK3B overexpression reversed these effects (Fig. 6A, C). Furthermore, TFR1 knockdown reduced the contents of intracellular total iron (Fig. 6D) and labile iron (Fig. 6E, F) in AD-iPS cell-differentiated neural cells, while GSK3B overexpression abolished these effects. Moreover, TFR1 knockdown inhibited ROS production, which was reversed by GSK3B overexpression (Fig. 6G). Additionally, TFR1 knockdown increased the MMP (Fig. 6H, I) and intracellular ATP content (Fig. 6J), while GSK3B overexpression reversed these effects. Mitochondrial staining showed that sh-TFR1 transfection improved mitochondrial fission and fragmentation, exhibiting typical length and tubular shape compared with sh-NC group, whereas GSK3B overexpression abrogated this effect (Fig. 6K). Moreover, the levels of DRP1 and FIS1 were reduced and the levels of MFN2 and OPA1 were increased by TFR1 knockdown, while GSK3B overexpression reversed these effects (Fig. 6J–L). Our results demonstrated that TFR1 modulated iron overload and mitochondrial dysfunction during AD-iPS cell differentiation through regulating GSK3B.

## Discussion

Iron is an essential element for cellular function and the maintenance of neuronal systems. However, neurodegenerative diseases such as AD and Parkinson's disease have been associated with iron metabolism disorders, particularly iron overload [25, 26]. Iron overload leads to neuronal toxicity through ROS production, oxidative stress, mitochondrial dysfunction, and inflammation [27, 28]. In this study, we established a precise human neural cell model of AD through inducing AD patient-derived iPS cells into neural cells in a 3D human neural cell culture system. During the differentiation process, we observed a significant upregulation of ferritin and ROS levels. Additionally, TFR1 protein expression was gradually elevated during AD-iPS cell differentiation. Thus, we speculated that TFR1-associated iron overload might participate in the regulation of AD progression.

Iron homeostasis alteration is closely related to mitochondrial dysfunction, which is implicated in the onset of AD characterized by amyloidosis and tau phosphorylation [29]. Numerous studies have illustrated that mitochondrial dysfunction contributes to AD pathogenesis through various mechanisms, such as abnormal mitochondrial gene expression, increased mitochondrial DNA mutation, decreased mitochondrial enzyme activity, defective mitophagy and altered dynamics [29–31]. Iron overload means an increase in intracellular iron influx and a decrease in iron efflux, is known to be mediated by TFR1 in neuronal cells and tissues [11]. Previous research has shown interventions targeting TFR1 expression can mitigate iron overload in different contexts. For example, liraglutide mitigated iron overload through decreasing TFR1 expression and increasing FPN1 expression in the livers of diabetic mice [32]. Salvia miltiorrhiza injection reduced cardiac iron accumulation and improved cardiac function by reducing cardiac iron uptake and increasing iron excretion through downregulating TFR1 expression and upregulating FPN1 expression [33]. Additionally, TFR1 knockdown reversed palmitate-induced iron overload and insulin resistance in human skeletal muscle cells [34]. Therefore, TFR1 is considered as a momentous target to prevent cellular iron overload and ferroptosis.

Additionally, mitochondrial dysfunction and cellular iron overload is closely associated with abnormal alterations in TFR1 expression [12]. Recent studies have shown that TFR1 knockdown decreases iron accumulation and maintains mitochondrial function inhibited malignant behavior in hepatocellular carcinoma-derived cancer stem-like cells [15]. Furthermore, Biochanin A alleviated mitochondrial damage induced by iron overload in chondrocytes through reducing intracellular iron level via inhibiting TFR1 expression [35]. More notably, it was previously proved that the mRNA and protein levels of TFR1 was higher in the cerebral cortex of transgenic mouse model of AD than C57 wild-type mice [13], indicating that abnormal TFR1 expression may contribute to the development of AD. Therefore, the present study investigated the effects of TFR1 on iron overload and mitochondrial function in AD progression. Our results demonstrated that TFR1 protein expression was upregulated during AD-iPS cell differentiation. TFR1 overexpression increased intracellular total iron and labile iron levels, as well as ROS production in AD-iPS cell-differentiated neural cells. Moreover, TFR1 overexpression facilitated mitochondrial fission, induced MMP loss, and reduced intracellular ATP content. In contrast, TFR1 knockdown attenuated iron overload, ROS production and mitochondrial dysfunction in AD-iPS cell-differentiated neural cells.

GSK3B is a glycogen metabolism enzyme that involved in several physiological processes from glycogen metabolism to gene transcription. It has been suggested that GSK3B homeostasis is closely related to the pathogenesis of neuropsychiatric diseases, and GSK3B activity was increased within the brain of AD patients [36]. Scientific evidence has proved that GSK3B regulates several histopathological hallmarks of AD, such as increased Aβ production, aberrant hyperphosphorylation of Tau protein, memory impairment, and neuronal loss [37, 38]. Consequently, GSK3B has been defined as a potential therapeutic target for AD. For instance, apigenin had a neuroprotection effect in AD rat model by inhibiting GSK3B-mediated hyperphosphorylation of tau protein [39]. Additionally, GSK3B knockdown restrained the expression of amyloid pathway genes, restored insulin signaling, and rescued cognitive impairment symptoms in AD rat model [40]. Notably, neurons differentiated from iPS cells derived from AD patients exhibited elevated levels of active GSK3B, Tau hyperphosphorylation, and amyloid levels [41]. Consistently, our study confirmed that GSK3B protein expression was gradually elevated during AD-iPS cell differentiation. Furthermore, our protein binding database analysis revealed a potential interaction between TFR1 and GSK3B, although this interaction has not been reported thus far. Our subsequent experiments validated that TFR1 could interact with GSK3B and promote GSK3B expression in AD-iPS cell-differentiated neural cells.

GSK3B has been revealed to be involved in the regulation of iron overload and mitochondrial dysfunction by regulating the nuclear factor erythroid 2-related factor 2 (Nrf2) [42]. For instance, GSK3B downregulation has been observed in breast cancer tissues, and GSK3B overexpression enhanced ferroptosis and ROS generation in breast cancer cells [43]. Inhibition of GSK3B reduced iron accumulation and ROS production, and ameliorated ferroptosis in lens epithelial cells [44]. Moreover, Hesperidin-mediated inhibition of GSK3B activity mitigated cognitive impairment, mitochondrial dysfunction and oxidative stress in AD mouse model [45]. In our study, we demonstrated that TFR1 could interact with GSK3B and promote GSK3B expression in AD neural cells. Rescue experiments further confirmed the effects of the effects of TFR1/GSK3B axis on iron overload in AD neural cells. We found that GSK3B overexpression reversed the inhibitory effects of TFR1 knockdown on iron overload, ROS production and mitochondrial dysfunction in AD-iPS cell-differentiated neural cells. Therefore, our results revealed that TFR1 modulated iron overload and mitochondrial dysfunction during AD-iPS cell differentiation partly through the regulation of GSK3B.

In summary, our findings revealed that TFR1 was upregulated during AD-iPS cell differentiation. TFR1 knockdown attenuated iron overload, ROS production and mitochondrial dysfunction in AD-iPS cell-differentiated neural cells. Mechanistically, TFR1 facilitated AD progression through interacting with GSK3B and promoting GSK3B expression. Our study may provide insights into a potential therapeutic target for AD.

## Data Availability

The datasets used during the present study are available from the corresponding author upon reasonable request.

## Abbreviations

AD: Alzheimer’s disease
NFTs: Neuronal fibrillary tangles
Aβ: Amyloid β
p-Tau: Phosphorylated Tau protein
ROS: Reactive oxygen species
TFR1/TFRC: Transferrin receptor
GSK3B: Glycogen synthase kinase 3 beta
FPN1: Ferroportin 1
FTH1: Ferritin heavy chain 1
ApoE: Apolipoprotein E
iPS: Induced pluripotent stem
EDTA: Ethylenediaminetetraacetic acid
AAS: Atomic absorption spectrometer
MMP/Δψm: Mitochondrial membrane potential
ATP: Adenosine triphosphate
APP: Amyloid precursor protein
DRP1: Dynamic related protein 1
FIS1: Fission 1
MFN2: Mitofusin 2
OPA1: Optic atrophy 1
